# Additive Manufacturing of Fe-Mn-Si-Based Shape Memory Alloys: State of the Art, Challenges and Opportunities

**DOI:** 10.3390/ma16247517

**Published:** 2023-12-05

**Authors:** Lucia Del-Río, Maria L. Nó, Raul Gómez, Leire García-Sesma, Ernesto Urionabarrenetxea, Pablo Ortega, Ane M. Mancisidor, Maria San Sebastian, Nerea Burgos, Jose M. San Juan

**Affiliations:** 1 Department of Physics, Faculty of Science and Technology, University of the Basque Country, UPV/EHU, P.O. Box 644, 48080 Bilbao, Spain; lucia.delrio@ehu.eus (L.D.-R.); maria.no@ehu.es (M.L.N.); 2 LORTEK-Basque Research Technology Alliance, BRTA, Arranomendia Kalea 4A, 20240 Ordizia, Spainlgarcia@lortek.es (L.G.-S.);; 3 CEIT-Basque Research Technology Alliance, BRTA, Manuel de Lardizabal 15, 20018 Donostia-San Sebastian, Spainportegar@ceit.es (P.O.); nburgos@ceit.es (N.B.); 4 Universidad de Navarra, Tecnun, Manuel de Lardizabal 13, 20018 Donostia-San Sebastian, Spain

**Keywords:** additive manufacturing, laser powder bed fusion, shape memory alloys, Fe-Mn-Si-Cr-Ni, martensitic transformation

## Abstract

Additive manufacturing (AM) constitutes the new paradigm in materials processing and its use on metals and alloys opens new unforeseen possibilities, but is facing several challenges regarding the design of the microstructure, which is particularly awkward in the case of functional materials, like shape memory alloys (SMA), as they require a robust microstructure to withstand the constraints appearing during their shape change. In the present work, the attention is focused on the AM of the important Fe-Mn-Si-based SMA family, which is attracting a great technological interest in many industrial sectors. Initially, an overview on the design concepts of this SMA family is offered, with special emphasis to the problems arising during AM. Then, such concepts are considered in order to experimentally develop the AM production of the Fe-20Mn-6Si-9Cr-5Ni (wt%) SMA through laser powder bed fusion (LPBF). The complete methodology is approached, from the gas atomization of powders to the LPBF production and the final thermal treatments to functionalize the SMA. The microstructure is characterized by scanning and transmission electron microscopy after each step of the processing route. The reversibility of the ε martensitic transformation and its evolution on cycling are studied by internal friction and electron microscopy. An outstanding 14% of fully reversible thermal transformation of ε martensite is obtained. The present results show that, in spite of the still remaining challenges, AM by LPBF offers a good approach to produce this family of Fe-Mn-Si-based SMA, opening new opportunities for its applications.

## 1. Introduction

Shape memory alloys (SMA) are functional materials that can recover their original shape after deformation, exhibiting the shape memory effect (SME), thanks to a reversible martensitic transformation, between the high-temperature phase called austenite and the low-temperature phase called martensite, which is dominated by a crystallographic shearing of the austenite lattice; see the textbooks for a general overview [1,2,3]. Thanks to their functional behavior, SMAs are widely used in different industrial sectors, e.g., civil and structural engineering, aerospace, automotive, biomedical and robotics; see the recent reviews [4,5]. Among the different families of SMA, Fe-Mn-Si alloys are attracting a renewed interest due to their high recovery stress during shape memory, enhanced damping capacity and low cost. Discovered in 1982 by Sato et al. [6,7,8] in a seminal series of papers on Fe-Mn-Si single crystals, and later by Murakami et al. [9] in polycrystals, the Fe-Mn-Si-based family evolved towards Fe-Mn-Si-Cr-Ni, to improve the corrosion resistance [10,11,12]. Because of the indicated properties, this family of SMA is becoming extremely suitable for numerous applications, particularly within the field of civil engineering [13,14,15,16,17,18,19,20,21,22,23,24,25,26,27,28]. This material exhibits a martensitic transformation from an FCC *γ* austenite to an HCP *ε* martensite, which occurs by the motion of a/6<112> Shockley partial dislocations on every two (111) close-packed planes of the FCC structure, according to the model of Olson and Cohen [29]. The motion of said partials, and therefore the nucleation and growth of the *ε* martensite, is fully reversible and is responsible for the SME of these alloys [30]. However, there is another alternative martensitic transformation from the FCC *γ* austenite to a BCC *α*′ martensite [31]. Nevertheless, contrary to the *ε* martensite, the concurrent *α*′ martensite represents a barrier for dislocation movement, hindering the back motion of the Shockley partials and deteriorating the SME [8,32]. Because of these intrinsic mechanisms of martensite nucleation and growing, the martensitic transformation of this family of SMA is non-thermoelastic [1,2,30], unlike the commonly used Ti-Ni- or Cu-based SMA [1,2,33,34], and the immediate consequence is that only a limited fraction of austenite transforms into martensite. Thus, the optimization of the transformed volume fraction of *ε* martensite becomes a key aspect for practical applications and many efforts are being devoted to the enhancement of the *ε* fraction through the composition and microstructure of Fe-Mn-Si-based alloys.

In parallel, additive manufacturing (AM) of metals emerged as a new paradigm of materials processing and has evolved very fast from prototyping to a real production technology in the recent years; see the reviews [35,36,37] for a general description of AM in metals and alloys. As an innovative fabrication process with short lead times, AM opens a new perspective to overcome constrains of conventional manufacturing in terms of complexity and to exploit the attractive functionalities of SMA, being already applied mainly to the production of TiNi [38,39,40], Cu-based [41,42,43] and Ni-Mn-based [44,45,46,47] SMA. In this context, research on the AM of Fe-based SMA has been only recently approached by laser powder bed fusion (LPBF) [48,49,50,51,52,53], laser metal deposition (LMD) [54] and wire arc (WAAM) [55], and the AM parameters must be still optimized in order to obtain the required microstructure to exhibit a good martensitic transformation on cycling, in order to guarantee the expected performances during shape memory and damping applications.

Then, the present work has a twofold objective. First, the state of the art of Fe-Mn-Si-Based SMA will be overviewed with special emphasis on the key points of the alloy design and the corresponding challenges for producing these SMA by AM. Second, in light of the previous analysis, the complete methodology for producing Fe-20Mn-6Si-9Cr-5Ni (wt%) SMA by AM will be developed, covering the full process, from gas atomization of the alloy to the AM through LPBF additive manufacturing, also known as the Selective Laser Melting (SLM) technique. A complete microstructure characterization by electron microscopy is carried out at the different steps of the processing route. The suitable thermal treatments are discussed, along with the corresponding microstructures and the study of the reversible γ-ε martensitic transformation, which is approached by mechanical spectroscopy. The thermal shape memory properties of the LPBF fabricated samples are compared with the performance of previously produced specimens by conventional casting. The SMA samples produced by LPBF exhibit a good martensitic transformation and better cycling evolution, stimulating further studies and paving the road for the development of new applications.

## 2. State of the Art and Challenges for AM of Fe-Mn-Si-Based SMA

The Fe-Mn-Si-based SMA constitutes an important family of the high-Mn steels, which also includes the Hadfield steels, the twinning induced plasticity (TWIP) steels and the transformed induced plasticity (TRIP) steels; see the review on the Fe-Mn-based alloys [56]. From the early works, it is known that high-Mn steels (from 10 to 30 wt% Mn) exhibit the ε martensitic transformation, particularly in extra-low carbon steels; see [31] for an overview of those early works. It is also known that Mn is a γ stabilizer and, consequently, the ε martensite start temperature Ms decreases when increasing the Mn content; this dependence was determined for Fe-Mn alloys [57] and for Fe-Mn-Si alloys [42]. However, below 14.5 wt% Mn, the two stage transformation γ-ε-α′ is promoted [31,57] and it has been largely observed that the apparition of α′ martensite deteriorates the shape memory behavior [8,30,32,58,59,60,61,62,63,64]; consequently, for the design of Fe-Mn-Si-based SMA, the Mn content should be above the 15 wt% in Mn. At this point, it must be indicated that in addition to both martensitic transformations ε and α′, the γ austenite also undergoes a magnetic transition from a paramagnetic state at high temperature to an antiferromagnetic state when cooling below the Neel temperature, T_N_, which stabilizes the γ phase, lowering its energy and inhibiting the martensitic transformation [7,57,65,66,67]. Indeed, the magnetic transition produces a dynamic modulus softening of the austenite γ preventing the ε martensite transformation, as was clearly shown in [65] and further confirmed in [66,67]. Thus, if T_N_<Mf (martensite finish) no interaction is expected between the magnetic and martensitic transformation, but, on the contrary, if T_N_>Ms the martensitic transformation may be fully inhibited by the magnetic transition, and partially inhibited when Mf<T_N_<Ms. Consequently, the alloy must be designed to have the T_N_ as low as possible. Thus, we have to face a serious quandary, because the Mn decreases Ms, but increases T_N_ [42,57]. Hence, one of the reasons to add Si to the Fe-Mn alloys is just to solve this problem, because Si strongly decreases T_N_ [42,68]. The other important reason for adding Si comes from the fact that Si decreases the stacking fault energy, generating large stacking faults between the two Shockley partial dislocations a/6<112> [69,70,71], in which the perfect dislocations of the austenite γ are dissociated, therefore constituting the nucleation places for the ε martensite lathes. Thus, the addition of Si is a key point for obtaining shape memory effect in this family of SMA, which increases linearly with the Si content [72]. Nevertheless, the Si content is limited to 6 wt%, because above this value the alloy becomes very brittle for further rolling [42,54,67]. Then, values between 5 and 6 wt% of Si are considered for the family of Fe-Mn-Si SMA.

Other alloying elements were considered, in particular Cr and Ni, to increase the corrosion resistance of the alloys [10,11,12]. Another advantage of Cr is that, unlike Mn, it decreases the T_N_ [73], whereas the influence on lowering Ms is smaller than the Mn [74], and contents between 5 and 12 wt% Cr are currently used in Fe-Mn-Si-based SMA [75]. Alloying with Ni, which is a strong γ-stabilizer, highly decreases Ms and increases the stacking fault energy [11], but on the positive side, it decreases T_N_, and helps the reverse martensitic transformation [76]. Nevertheless, above 6 wt% Ni promotes the generation of mechanical twins suppressing the strain-induced ε phase. For these reasons, only 4 to 5 wt% Ni is currently used in Fe-Mn-Si-based SMA. Cobalt is also being added as an alloying element, because it also crystallizes in the hexagonal ε phase [31] and it was expected that could help the development of the ε martensite in Fe-Mn-Si-based SMA, but the influence of Co is a source of controversy. Indeed, Co slightly decreases T_N_ [77,78,79] as well as the stacking fault energy [80]. In one of these works [79], with 6 wt% of Co or Si, the authors concluded that Co has a reduced improvement of the shape memory effect with respect to Si. On the contrary, in a previous work [80] with a smaller amount of Co, the authors showed a noticeable increase in the shape memory recovery reaching a maximum between 3 and 4 wt% Co. Taking into account that the content of Si is, in practice, limited to 6 wt%, the simultaneous addition of such a small amount of Co may offers a supplementary reduction in T_N_, contributing to the formation of a higher fraction of ε martensite.

The influence of interstitial atoms, carbon C, nitrogen N and oxygen O, has been scarcely studied and there are some discrepancies on their effect on the shape memory behavior. Very often, these elements appear as impurities coming from the precursor master alloys, or are introduced during processing. From the early works on Mn-rich steels [31], it was recommended that an extra-low C content could improve the development of ε martensite. Indeed, C has only a slight influence on T_N_, but a strong influence on decreasing the Ms in Fe-Mn steels [68] and, consequently, prevents the ε martensitic transformation. Furthermore, in Si-rich alloys, C is depleted from the solid solution of austenite and compromises the chemical homogeneity of the alloy [81]. In general, C has a detrimental effect on the SME of Fe-Mn-Si-based SMA [75]. Some authors [82] claim that the shape recovery in C-bearing Fe-Mn-Si-based alloys is improved when the deformation during pre-straining is performed at a low temperature (77 K), although it is smaller when deformed at room temperature. In our opinion, this experimental fact could have a simple explanation. Due to the influence of Si, C is segregated from the austenite solid solution and diffuses towards the core of the dislocations, creating Cotrell or even Snoek atmospheres around or over the stacking faults. In this scenario, C atoms would anchor the leading Shockley dislocation, preventing the progress of the ε transformation. During deformation at room temperature, C diffuses easily and the Cotrell atmosphere moves with the faulted dislocations. On the contrary, when pre-deforming at a very low temperature (77 K), C atoms cannot diffuse fast enough to follow the dislocation and a breakaway process takes place; the dislocation becomes free of C and the leading Shockley partial can easily move forward, allowing the growth of ε martensite. The negative influence of C is extremely important because, in many cases, metals and master alloys used for processing may contain a noticeable amount of C > 0.1 wt%. There are several authors that utilize second phase precipitation to strength the austenite matrix, using carbide forming elements like Nb, V and Ti to precipitate the corresponding carbides: NbC, VC, TiC or even Cr_23_C_6_ carbides. However, there is no general agreement on the reason for the SME improvement; see [75] for an overview. In our opinion, this effect could be attributed not only to the precipitation strengthening, but also to the depletion of C from the solid solution to form the precipitates. This precipitation cleans up the lattice of austenite from C atoms and the Shockley partials become free for moving, allowing the progress of the ε martensitic transformation.

There are few works about the influence on SME of other interstitials like nitrogen or oxygen. Apparently, N does not affect the chemical homogeneity of the solid solutions in this kind of alloys and helps to suppress the apparition of α′ martensite during quenching [81]; so, no detrimental effect is expected at the level of impurities. The influence of oxygen has not been seriously studied and constitutes an open topic, which may be particularly relevant in the case of AM-processed alloys, as we will comment on within the experimental section.

In light of the above analysis, several challenges can be identified for AM processing of Fe-Mn-Si-based SMA:

- First, porosity must be reduced to obtain a fully compact alloy. This is a general requirement for the AM of alloys, but it is particularly relevant for SMA, in order that the microstructure will not be degraded by the local stresses associated with the phase transformation and its changes on cycling.
- Second, it becomes necessary to accurately master the concentration of each element of these complex alloys, because, as was previously discussed, the optimization of the properties requires very narrow ranges of each element concentration. This matching must be guaranteed along all processing routes, from atomization to the pool melting during AM.
- Third, in relation to the previous point, the control of the critical elements that may evaporate or react with crucibles generate important losses, in Mn or Si for instance, must be compensated. The use of pure metals and clean master alloys is strongly recommended.
- Fourth, the control of impurities, coming from the rough materials or unintentionally introduced during processing, must be a subject of special care. A relevant aspect for AM is the oxygen, because powders with an oxidized surface are being used.

To face these challenges, a clever design of the AM parameters must be approached: in particular, laser power and scanning speed. The useful magnitude summarizing the influence of several parameters is the volumetric energy density (VED) defined in Equation (1). Here, it is worthy to note that Fe-Mn-Si-based SMAs share some problematic aspects with Ni-Mn-based SMAs because both kind of alloys contain a high amount of Mn, which is a highly volatile element prone to important losses. From the literature of AM in these alloys, some lessons can be learned: for VED < 55 J/mm^3^, samples are not properly compacted exhibiting high porosity and low densities [44,48]; on the contrary, for VED > 145 J/mm^3^, important losses of Mn, above 6 wt%, are reported [45,52]. Obviously, these limit values are strongly modified in case of some preheating being applied on the powder bed platform, as recently reported [47]. 

Another important aspect for the design of the final SMA comes from the intrinsic behavior of the ε martensite nucleation in the stacking faults in between the dissociated Shockley partial dislocations. This fact highlights a key difference between the conventional processed samples and the AM processed ones. The conventional processing route of Fe-Mn-Si-based SMA involves casting and further hot rolling (or forging), very often finishing with a cold rolling. During these deformation processes, a high-density network of dislocations is developed, and even after recrystallization, the material contains a high density of dislocations, which constitutes the nucleation points for ε martensite. Then, in order to obtain the maximum transformed fraction from γ austenite to ε martensite, numerous works have been devoted to the study of the thermomechanical treatments more appropriate to obtain an optimum SME and to control the microstructure, including aspects such as precipitation, grain size and texture, among others [83,84,85,86,87,88,89,90,91,92,93]. A review on this broad topic is out of the scope of the present paper but it must be remarked that this is a point in which AM exhibits less degree of freedom on the design due to its specific processing route. Indeed, when processing through AM, it is supposed that samples will not undergo further heavy deformation, as in rolling or forging. Instead, the available variables come from thermal treatments, or eventually, from the control of internal stresses through the heating and cooling rates during AM processing. The control of aspects such as the grain size or texture, traditionally more or less well-controlled by the conventional processing routes, are more arduous to control by AM and constitutes a challenge (for AM) in order to optimize the microstructure for a maximum SME in this kind of SMA.

## 3. Materials and Methods

### 3.1. Powder Atomization

With these criteria in mind, and in order to obtain a Ms close to room temperature, the selected composition of the alloy for the production through AM was Fe-20Mn-6Si-9Cr-5Ni (wt%). Powders of the alloy were produced by gas atomization due to its high productivity, reasonable process control and the possibility to produce spherical particles, with good packing characteristics, which are optimal for their use in AM [35,36,37]. The process was carried out at the CEIT technology center in a small–scale research atomization unit PSI model HERMIGA 75/3VI (Phoenix Scientific Industries Ltd., Hailsham, UK); a description of the equipment is presented elsewhere [94]. High purity (>4 N) alloying elements were used, except for the Cr that was added as a Fe-Cr master alloy. High-purity Argon was used for both the melting process and the atomization at a pressure of 1.73–1.75 MPa (gas mass flow rate of 53.5–58 g/s). An overheating of around 180 K was used above the melt temperature, which, in the furnace, corresponded to 1733 K. The amount of powder produced per atomization is 3 kg, and three atomizations were necessary to obtain the quantity of powders required for the LPBF additive process. 

**Figure 1:**
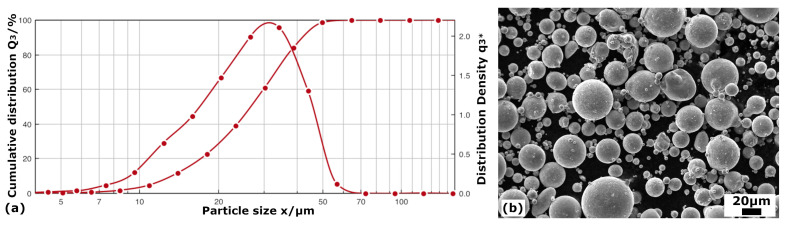
(**a**) Particle size distribution: frequency and cumulative integral for the atomization process of the Fe-based SMAs; (**b**) SEM micrography of the atomized powders with a medium size of 20–45 μm.

After the atomization, the particle size distribution was measured by dynamic image analysis in a Sympatec QICPIC equipment (Sympatec GmbH, Clausthal-Zellerfeld, Germany), and the result is presented in Figure 1a. In addition, the powders were analyzed by ICP (Inductively Coupled Plasma) in a Varian 725–ES ICP–OES equipment (Agilent Technologies, Santa Clara, CA, USA). Once the composition of the powders from the three atomizations were verified to be homogeneous, all powders were mixed, resulting in a final composition of Fe-19.4Mn-5.9Si-9.2Cr-5.1Ni (wt%). Finally, sieving was also carried out to isolate the fraction of spherical powders between 20 and 45 μm in diameter, as required for the LPBF used technique; thus, the retained fraction turned out to be about 60% of the atomized powders. The quality of the finally used powders is shown in the scanning electron micrographs of Figure 1b.

### 3.2. LPBF: Selective Laser Melting

The powders were processed by LPBF technology using a Renishaw AM 500Q (Renishaw plc., New Mills, UK), with dynamic focusing of four ytterbium fiber lasers with a spot laser of 80 μm. For greater efficiency, also used was the so–called reduced build volume (RBV) module, which allows the construction of AM samples with small amounts of metal powder (from 2 to 4 Kg). 

The proposed design of experiment (DoE) consisted of the fabrication of cubic samples of 10 × 10 × 10 mm^3^ using different sets of parameters that, taking into account the previous comments, corresponds to the low intermediate region of VED, in order to minimize the Mn losses. With a constant value of 120 μm for the hatch distance *h* and a layer thickness *t* of 30 μm, a series of samples with different experimental conditions were obtained by varying the scanning speed *v* and the power *P* of the laser. The volumetric energy density (VED), *E*, can be expressed as a function of the previously mentioned parameters in accordance with the following Equation (1) [35,95], and are listed in Table 1 for all of the DoE samples.

(1) $$E=\frac{P}{v\cdot h\cdot t}$$

**Figure 2:**
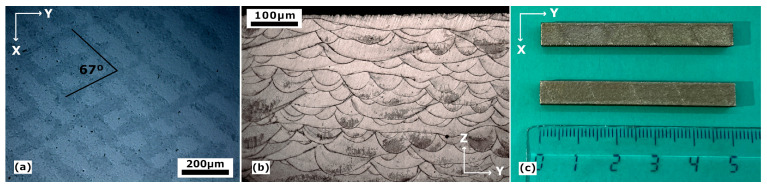
(**a**) OM image of the XY plane LPBF processed sample where it is shown the 67° stripes scanning strategy; (**b**) transversal OM image of the YZ plane LPBF processed sample along the build direction; (**c**) mentioned LPBF processed sample (rule numbers are cm).

The scanning strategy also has a great influence on porosity and the final consolidation of the samples, for what the so-called stripes at 67° strategy was chosen, Figure 2a. Moreover, in Figure 2b, shown is the microstructure along the scanning build direction (YZ plane) of the samples, in which we can find the characteristic melt pool geometry of the LPBF-processed samples. It can also be noted that, due to the laser passes at 67°, narrow and wider pools alternate when looking along the build direction. The compactness of each sample was measured by image analysis using the software SW Analysis Docu (PAQXOS V 5.0), which consists of measuring the area of the corresponding defects with respect to the total area of the images.

These images were taken from a cut on the XY plane of the cubes, that is, perpendicular to the build direction. The optimized parameters for the most compact sample were those with the highest power, 200 W laser power and 650 mm/s scanning speed, which results in a VED of 85 J/mm^3^. Finally, with a set of dimensions of 5 × 5 × 60 mm^3^, the massive material fabricated with those parameters is shown in Figure 2c; this is the sample used for subsequent studies.

### 3.3. Microstructural Characterization Methods

After the LPBF, the processed samples were cut by electrical discharge perpendicular to the build direction and a microstructural characterization was conducted via electron microscopy techniques. The specimens were prepared by conventional mechanical polishing down to a particle size of 1 μm in a Struers Abramin (Struers Tech A/S, Rodovre/Copenhagen, Denmark). Afterwards, the samples were electro-polished (Struers ElectroPol-5) with a solution of 700 mL of ethanol, 100 mL of glycerin and 200 mL of HClO_4_, with 10–12 V up to 180 s at room temperature. On the other hand, in order to distinguish the as-built microstructure at the optical microscope (Leika DMRXA 2, Leica, Cambridge, UK), a chemical attack was performed with a solution consisting of CuCl_2_ (1 g), HCl (50 mL), HNO_3_ (50 mL) and H_2_O (150 mL) for 3 s. Additionally, the samples were cleaned in an Ar-25%O_2_ plasma cleaner (FISCHIONE, model 1020, FISCHIONE Instruments Inc., Export, PA, USA) before the scanning electron microscopy (SEM) observation. The SEM study was conducted on a focused ion beam (FIB) (FEI Helios NanoLab650, FEI, Hillsboro, OR, USA) equipped with backscatter electrons (BSE) and Energy Dispersion X-ray spectroscopy (EDX) (Oxford X-MAX, Oxford Instruments, Abingdon, UK) detectors, and in a Schottky SEM (JEOL JSM-7000F, JEOL Ltd., Tokyo, Japan) equipped with BSE, EDX (Oxford inca350) and electron backscatter diffraction (EBSD) (Oxford CMOS) detectors, with five forescatter diodes (FSD) to obtain the image of the tilted surface of the sample. Unless expressly indicated, the BSE experimental conditions were 20 kV, 1.6 nA, and 10 kV, 0.8 nA, for the FEI and JEOL equipment, respectively; 20 kV and 1.6 nA for the compositional analysis and 20 kV and 1 to 5 nA for the EBSD. Moreover, a Zeiss SEM Ultra Plus (Zeiss, Jena, Germany) with Oxford Nordlys detector at 15 kV was also used for EBSD characterization.

The transmission electron microscopy (TEM) characterization was performed in a Phillips CM-200 with a double tilt sample holder operating at 200 kV. In this case, the samples were flattened and thinned until around 100–75 μm foils and jet-polished, using a 70% ethanol, 10% glycerin and 20% perchloric acid solution, in a double-jet Struers TenuPol-5 with 14 V at 258 K. In addition, Scanning Transmission Electron Microscopy (STEM) was performed at 300 kV on a FEI Titan Cubed G2 60–300 kV (FEI, Hillsboro, OR, USA) with a gun monochromator, a Cs-objective aberration corrector (CEOS) and super-X EDX detector.

### 3.4. Phase Transformations Characterization Techniques

As previously commented, in these Fe-Mn-Si-based alloys, the austenite undergoes, during cooling, a magnetic transition from a paramagnetic to an antiferromagnetic state, at the Neel temperature, T_N_, which stabilize the austenite and inhibits the martensitic transformation [52,60,61]; thus, it is important to determine T_N_ and reassure that it is lower than the martensite finish temperature, Mf. For this task, the magnetic susceptibility of the LPBF sample was measured in a PPMS Quantum Design magnetometer (Quantum Design North America, San Diego, CA, USA) and compared with an as-cast reference sample measured in a SQUID MPMS-7 T (Quantum Design). The results are presented in the Supplementary Information, Figure S1, and shows a T_N_ = 109 K for the LPBF sample.

The study of the martensitic transformation behavior was approached by internal friction (IF) and dynamic modulus (DM) measurements. The equipment used is a subresonant inverted torsion pendulum, homemade equipment [96], working under a low-pressure (4 mbar) Helium atmosphere in a temperature range between 100 K and 1150 K, with a frequency range from 10^−3^ to 5 Hz. The strength, Δ, of the IF peak associated with the martensitic transformation depends on frequency and on the heating–cooling rate ($\Delta \approx \dot{T}/\omega$) [97], so a constant heating–cooling rate of 1.5 K/min and a frequency of 0.5 Hz were used for all measurements, as well as a constant strain amplitude of ε_o_ = 2 × 10^−5^. IF measurements were performed in the temperature range from 100 K to 510 K, so that it comprised the whole interval Mf-Af of the forward and reverse martensitic transformation. Nonetheless, it must be mentioned that the cooling rate of 1.5 K/min cannot be kept when approaching 100 K, breaking the relationship between the IF peak and the transformed fraction during the direct transformation. However, it is demonstrated [97,98,99] that the integral of the IF peak during the forward transformation on cooling is equal to the one measured during the reverse transformation on heating; thus, the calculations of the transformed fraction were carried out, utilizing the data of the reverse transformation. For the IF measurements, samples of approximately 40 × 5 × 0.8 mm^3^ were cut from the massive LPBF material presented in Figure 2c.

## 4. Experimental Results

The development of a material through a new additive manufacturing method, like the LPBF, always requires a detailed microstructure characterization along the different processing steps, including the thermal treatments applied to control the microstructure. In the present work on Fe-Mn-Si-Cr-Ni SMA, this characterization is approached in Section 4.1. Then, the martensitic transformation (MT), responsible for the functional behavior of the SMA, is studied in Section 4.2 and its further cycling behavior in Section 4.3. The analysis of the experimental results will be simultaneously presented, before providing an overall discussion in Section 5.

### 4.1. Microstructure Characterization

With the purpose of obtaining the optimum and reversible martensitic transformation mass fraction, the material must accomplish homogeneity, not only compositional-wise, but also microstructural-wise, which is not achieved during the LPBF process. Indeed, the martensitic transformation requires, as the starting phase, the FCC γ austenite; this means that, as it will be shown, the additive manufactured samples should undergo a post-processing high temperature thermal treatment [83,84,85]. However, the temperature of the annealing treatment is controversial. Some authors [83,85] state that recrystallization annihilates the nucleation points for the martensite, so the heat treatment should be performed below the recrystallization temperature, while others [100,101] assure that an annealing above 1173 K or even 1273 K results in a higher amount of martensite due to the enlarged grains after recrystallization. With these precedents in mind, in the present work, two post-processing thermal treatments are considered, namely, 1200 K and 1350 K; in both cases the treatment consists in one hour in argon atmosphere and quenching in iced water.

#### 4.1.1. As-Built Microstructure

With the highest density of energy used in the present work (see Table 1) corresponding to the selected parameters of power P = 200 W and scanning speed v = 650 mm/s, the achieved compactness of the samples is 99.93%, with only some few processing defects. In similar samples, Ferreto et al. [44] reached a relative density of approximately 99% with P = 175 W and v = 600 mm/s, but enounced that the compactness could be increased reducing the scanning speed. However, the Mn content, which evaporates significantly during LPBF [102], does so even more when decreasing the scanning speed [47]. Therefore, it can be concluded that the final parameters chosen in the present work, have come to be a good equilibrium regarding compactness and Mn loss. 

**Figure 3:**
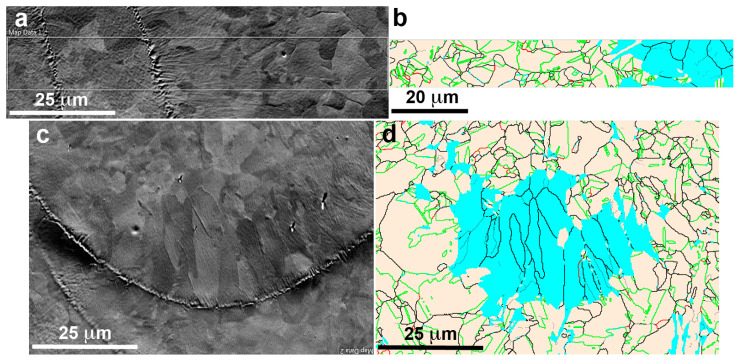
FSD images of the unspoiled LPBF sample, along with their corresponding EBSD phase map; (**a**,**b**) in the XY plane (EBSD step size 100 nm); (**c**,**d**) in the build-direction YZ plane (EBSD step size 200 nm). In both cases, ferrite is presented in blue and austenite is presented in light beige. Grain boundaries are presented in green (Σ3), red (Σ9) and black (the remaining ones).

Depending on the laser parameters and scanning strategy, the locally obtained temperature gradients and solidification rates will vary and the grain morphology will change in size and orientation, and an evolution of the present phases, or of its mass fraction, may also occur. Thus, the surfaces perpendicular and parallel to the build direction (Z) have been characterized. The BSE image of Figure 3a corresponds to the XY plane (equivalent to Figure 2a but just polished without chemical attack), where the grains are clearly seen, as well as two traces of the melting stripes. The EBSD image of Figure 3b, corresponding to the indicated rectangle in Figure 3a, allows the identification of the FCC γ-austenite phase (Fm-3m, a = 0.3615 nm), in light beige, and the BCC δ-ferrite phase (Im-3m, a = 0.2884 nm), in blue. The grain boundary surfaces in austenite have also been identified, mainly as ∑3 (<111>/60°) (42.3%) and ∑9 (<110>/38.9°) (2.7%) interfaces, according the coincidence site lattice (CSL) nomenclature [103,104]. Apparently, the δ ferrite appears in the regions where the grain size has grown, probably due to the secondary slow cooling during the superposition of adjacent melting stripes. The surface parallel to the build direction (YZ plane) is presented in the BSE image of the Figure 3c, equivalent to the Figure 2b, and the region of a solidified laser melted pool is clearly seen. This image shows the trace of the pool limit and the difference on the grain size and shape between the small equiaxed grains at the top, with a mean size of 4 μm, and the large columnar grains at the bottom, with a mean length about 20 μm.

On the right, the EBSD image of the same region, Figure 3d, shows the austenite grains in light beige (76.2%) and the ferrite grains in blue (23.8%). The comparison of such images, Figure 3c,d, evidences that the ferrite phase was developed at the bottom of the melt pool where the material solidify slowly given place to columnar grains, which are mainly of δ ferrite. A similar study on this build surface, including the grain orientation, is presented in the Supplementary Information, Figure S2. The analysis of these two surfaces evidences that in the LPBF build material, there is a coexistence of phases, the γ austenite and the δ ferrite, in good agreement with previous results in similar alloys [48,49,50,51,52,53]. Indeed, in a recent work at many different VED [105], it is shown that the presence of ferrite cannot be suppressed. Moreover, when Cr_eq_/Ni_eq_ is higher than 1.5, the precipitation of the BCC phase is greatly promoted [106]. So, considering that in our alloy the ratio Cr_eq_/Ni_eq_ is 2.17, it is well assumed that we will indeed have the presence of δ ferrite. Thus, it can be concluded that a further thermal treatment on the build samples will be required to obtain solely the austenite, from which the further transformation into ε martensite can occur.

#### 4.1.2. Microstructure after Thermal Treatment at 1200 K

**Figure 4:**
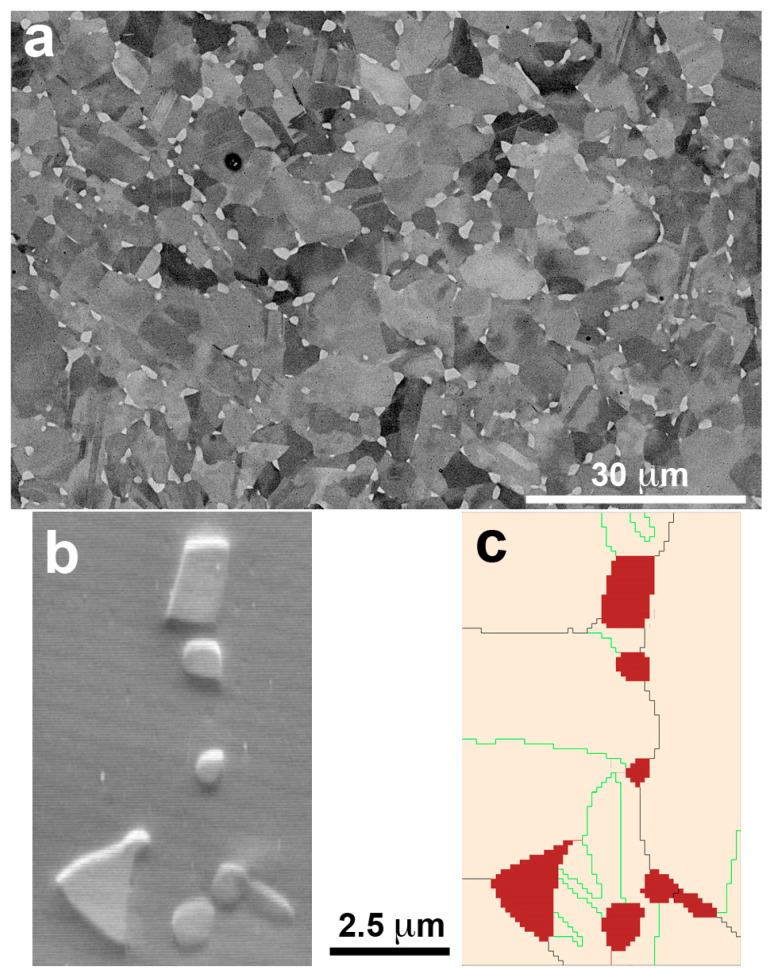
(**a**) SEM (BSE) image of the LPBF sample, showing the coexistence of two phases after the 1200 K heat-treatment. (**b**) FSD image of the same sample, along with its corresponding (**c**) EBSD map; βMn phase is in dark red, whereas the austenite is in light beige. Grain boundaries are in green (Σ3), red (Σ9) and black (the remaining ones).

From the previous section, the need of a thermal treatment to avoid the presence of δ ferrite becomes evident. However, to attain an optimum shape memory, a preexisting and uniformly arranged array of stacking faults through the matrix is needed [83,84,85]. Moreover, a single-phase material is required, so the movement of the martensite variants when growing is not inhibited. Therefore, even though the nucleation point may be reduced, a first treatment at 1200 K during one hour in argon atmosphere and quenching in iced water is proposed. Figure 4a shows a BSE micrograph where the existence of an additional phase is clearly appreciated with a lighter grey color, extensively precipitated at the grain boundaries. Quantitative EDX analysis shows that these precipitates are poorer in Fe than the matrix, with a composition Fe-20.0Mn-8.2Si-11.2Cr-8.0Ni (wt%). In order to identify this remaining phase after the thermal treatment, an EBSD study was carried out in several groups of precipitates. Figure 4b shows the FSD image of some selected precipitates and in Figure 4c the corresponding EBSD, in which the red phase is indexed as the β-manganese phase (P4_1_32, a = 0.63145 nm). 

According to the ternary Fe-Mn-Si phase diagrams [107,108], 1200 K is a high enough temperature to obtain a single FCC phase. However, there is a lack of information about the quinary phase diagrams, which are modified by the presence of Cr and Ni. A slight shift on the boundaries of the phase stability domains could explain the presence of these precipitates of β-Manganese phase. In addition, this result indicates that the alloying elements, Cr and Ni, have a significant effect on the recrystallization temperature, making it higher than expected. Indeed, the grain size in Figure 4a remains irregular with a mean value below 10 μm. Thus, it becomes obvious that a higher temperature is required for the thermal treatment.

#### 4.1.3. Microstructure after Thermal Treatment at 1350 K

Hence, the next step was to perform a thermal treatment at higher temperature, 1350 K. This temperature was chosen based on the previous literature [109], which reported that the single-phase austenite only becomes thermodynamically in equilibrium over 1273 K. 

**Figure 5:**
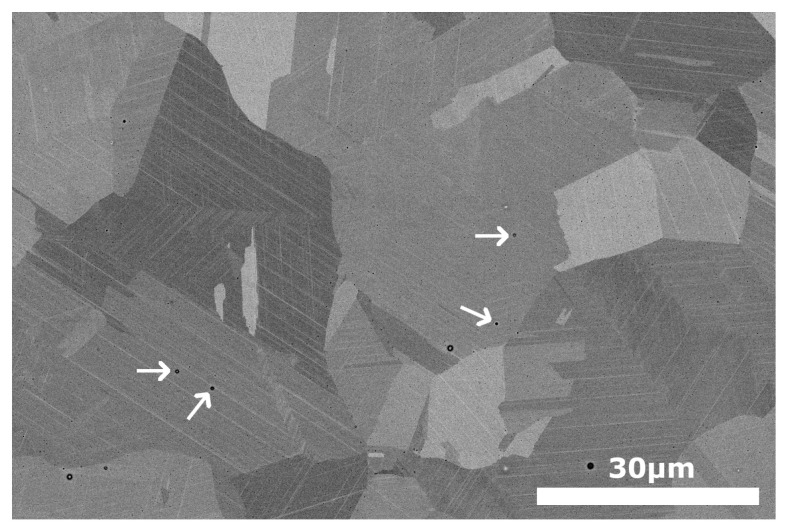
SEM (BSE) image of the LPBF sample, just after the 1350 K heat treatment, without any further thermal cycle. The thin white plates are the remaining martensite plates produced during the mechanical polishing. White arrows mark very small precipitates, which are examined by STEM-HAADF in Figure 6.

After the treatment at 1350 K, the BSE micrographs confirm the absence of the previous precipitates and the presence of a single austenite phase. A general view of the microstructure at low magnification is presented in the Supplementary Information, Figure S3, and the image of Figure 5 shows the microstructure at the same magnification as the one used in Figure 4a for comparison. The grain size has grown until an average size of 30 μm, and the matrix composition has become homogeneous. Nonetheless, it must be noted that the compositional analysis differed slightly from the ICP of the original powders. This leaves a final composition of the samples Fe-17.3Mn-6.1Si-9.4Cr-5.3Ni (wt%). As expected from the previous literature [106], there has been a loss of Mn during the LPBF process of approximately 2–3%. It is worth mentioning that the Mn loss expected during the atomization was already compensated, but, as commented on in Section 2, this result evidences that it is of capital importance to evaluate the losses along all steps of the processing route. Moreover, some very small precipitates, which at a first sight could be mistaken with pores, were seen along the sample, marked with white arrows in Figure 5. Since their size was too small to be characterized by SEM, a detailed observation was carried out by STEM.

**Figure 6:**
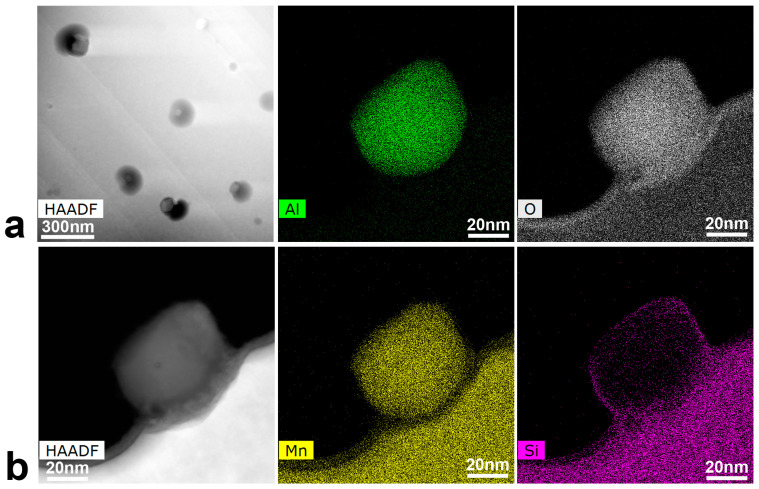
STEM at 300 kV. (**a**) HAADF image of the very small precipitates observed by SEM (marked by white arrows in Figure 5); (**b**) HAADF of a precipitate in the edge of the sample along with its corresponding EDX maps (I_beam_ = 0.25 nA) for element identification.

Figure 6a shows the STEM High-Angle Annular Dark Field (HAADF) image of such precipitates. However, taking into account that these precipitates are smaller than the thickness of the TEM lamella (<90 nm), the attention was focused on several precipitates close to the edge of the sample, Figure 6b, in order to do a quantitative analysis. 

The EDX maps of the precipitate in Figure 6b are presented on the right for the main elements, and show that those precipitates are composed by Al, O and Mn, with a thin layer of Si segregated to its outline. The composition from the EDX maps is Fe-17.2Mn-6.6Si-9.8Cr-5.6Ni (wt%) for the matrix, and 60.8O-26.3Al-11.5Mn (at%) for the precipitates, which leaves us with oxides of (Al,Mn)_2_O_3_ type. Although the apparition of such kind of precipitates during LPBF was not reported yet in these alloys, it has been considered as a general problem [110]. According to Jägle et al. [111], the precipitation happens during the deposition of the material, furthermore, due to the pronounced re-heating and reduced cooling rates of the previous layers, the precipitates are frequently induced. Regarding the composition, the presence of Mn oxides is also usual. Kraner et al. [112] reported that manganese is liable to oxidate during the atomization of the powders, so the oxygen is carried into the matrix, along with the Mn, forming Manganese oxides that do not dissolve into the matrix. In our case, the Aluminum, which comes from the impurities of the master alloys, just happens to replace de role of the Mn (Mn_2_O_3_), leading to those mixed oxides of (Al,Mn)_2_O_3_ type. This is an interesting point, because the Al, that is an undesirable impurity, serves the purpose of cleaning up the lattice from interstitial oxygen, even though some Mn is also lost from the solid solution.

All in all, it can be concluded that the desired microstructure was achieved with this last treatment and the following results are based in samples thermally treated at 1350 K.

### 4.2. Martensitic Transformation

**Figure 7:**
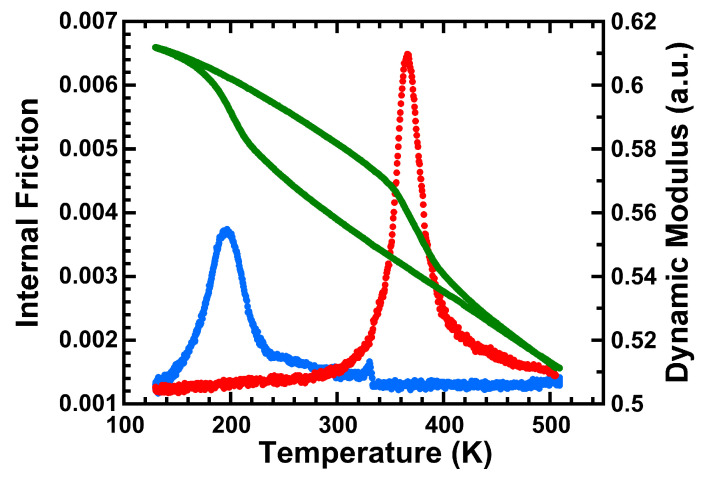
Internal friction spectra and dynamic modulus (green) measurements as a function of temperature for the heat-treated (at 1350 K) LPBF processed sample, in its 8th thermal cycle. In blue, the forward martensitic transformation on cooling and, in red, the reverse martensitic transformation on heating.

The internal friction spectra and the dynamic modulus curves for a complete transformation cycle are presented in Figure 7. The internal friction peaks correspond to the forward and reverse martensitic transformations and are associated with an abrupt change in the dynamic modulus (green). On cooling (blue), the direct or forward transformation from austenite to martensite occurs between Ms (279 K) and Mf (157 K) temperatures. On heating (red), the reverse transformation, from martensite to austenite, happens between As (324 K) and Af (458 K) temperatures. All temperatures are determined for a specific amount of the transformed mass fraction, calculated from the integral of the IF peaks [97,98,99], in our case 5% and 95%. 

The temperature range between the maximum of the forward and reverse phase transitions defines the thermal hysteresis, which results in a significantly high value of around 170 K, in good agreement with what is expected from a non-thermoelastic SMA. It must be mentioned that the dynamic modulus variation also exhibits a hysteresis similar to the one of the IF peaks. Moreover, it is noted that the dynamic modulus increases during the transformation, being associated with a hardening of the microstructure proportional to the transformed fraction, since ε has a higher modulus than γ. On the other hand, according to our previous studies [54,65], at low temperatures there is a modulus drop at the T_N_ temperature that corresponds to the magnetic transition. The fact that there is no such drop in our measurements, confirms that there is no interaction between the magnetic and martensitic transformations.

**Figure 8:**
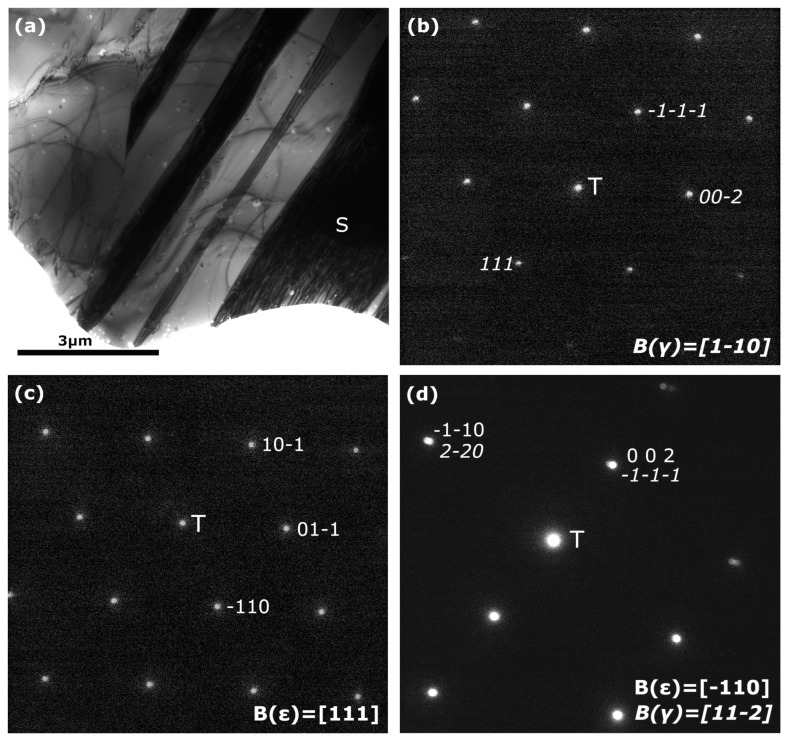
(**a**) TEM bright field image of a LPBF sample with one thermal cycle; (**b**) SADP pattern for the γ austenite matrix (T, transmitted beam); (**c**) Nano-diffraction pattern of the labeled S variant of ε martensite; (**d**) Nano-diffraction pattern for another ε variant surrounded by the austenite obtained for a different sample tilt. Both phases follow the Shoji-Nishiyama relationship.

In parallel to the IF measurements, a direct identification of the ε martensite was carried out by transmission electron microscopy. In Figure 8a, a bright field TEM image, where the dark martensite plates in diffraction condition are clearly contrasted over the matrix, is presented. Figure 8b shows a selected area diffraction pattern (SADP) obtained from the matrix. As expected, this diffraction pattern was indexed as the FCC γ austenite (Fm3m, a = 0.3615 nm). The dominant existence of this phase was verified in different sites of the sample. Figure 8c exhibits the nano-diffraction pattern obtained from the plate labeled S in the BF image. The diffraction pattern was indexed as the hexagonal ε martensite (P6_3_/mmc, a = 0.2555 nm, c = 0.4145 nm). 

Figure 8d shows, for a different sample tilt, the nano-diffraction pattern of another martensite variant inside the austenite phase and the indexation of this pattern corresponds to the coexistence of γ austenite and ε martensite, in good agreement with the Shoji-Nishiyama relationship [31].

**Figure 9:**
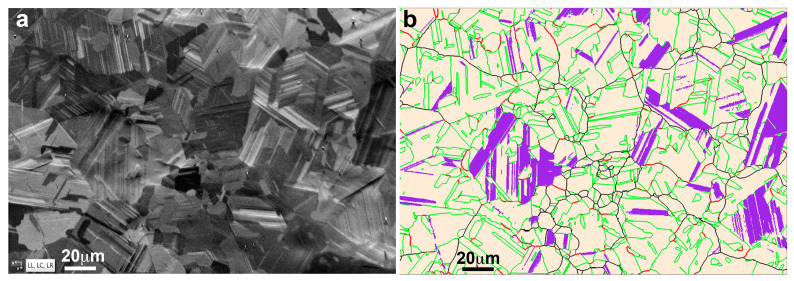
(**a**) FSD image of the heat-treated LPBF sample after 3 thermal cycles; (**b**) Corresponding EBSD phase map. The austenite is represented in light beige and martensite in purple (EBSD step size 240 nm). Grain boundaries are presented in green (∑3), red (∑9) and black (the remaining ones).

On the other hand, it is known that the α′ martensite is usually found in the intersection of ε martensite variants [8] and has a detrimental effect on the shape memory effect [32]. However, it must be mentioned that α′ martensite was not found during the TEM characterization. These results were also confirmed by the X-ray diffraction measurements presented in the Figure S4 of the Supplementary Information. Finally, an EBSD characterization was conducted on an equivalent sample after one thermal transformation cycle. In Figure 9, a FSD image and its corresponding EBSD image of the coexisting phases, γ in light beige and ε in purple, are presented. The grain boundaries of the γ austenite were also analyzed; the ∑3 (<111>/60°) interfaces are drawn in green and the ∑9 (<110>/38.9°) in red, according to the standard nomenclature [103,104], and the rest in black. As it can be appreciated when comparing both images, some martensite plates are not detected even with a step as small as 0.24 μm. This is because the thermal martensite develops by the nucleation and propagation of elementary ε layers with a few atomic planes [113]. Moreover, the thickness of the plates is not due to lateral growth, but the stacking of new ε layers grown besides these phases [114]. That is, ε thermal martensite lathes are extremely narrow in comparison with the stress-induced ones, and frequently are not thick enough to be indexed by the software, and, consequently, the resulting ε fraction obtained by EBSD (AZtechCrystal) is underestimated. Even so, the calculated fraction from Figure 9b for the third thermal cycle is around 12% in average of ε martensite. This is a good result, taking into account that maximum values of 10% were reported in similar alloys produced by conventional casting and rolling [101]. Moreover, the ε thermal martensites growth in each grain have mainly the same orientation minimizing their intersections and consequently avoiding the formation of α′ martensite, which in fact has not been observed. This means that it could be expected a good behavior when the stress-induced ε martensite be produced by strain during the SME; this approach is out of the present study and will be the subject of further works.

### 4.3. Thermal Cycling Behavior

**Figure 10:**
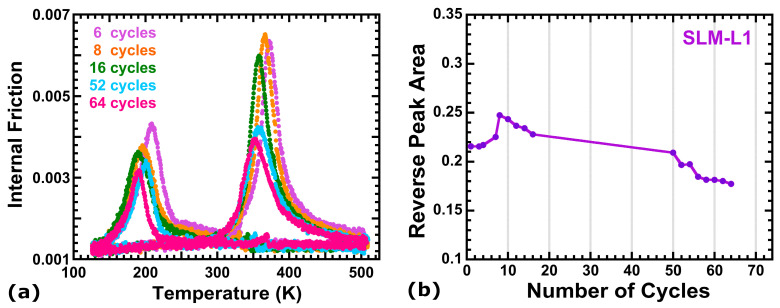
(**a**) Evolution of the internal friction spectra on cycling for the heat-treated LPBF alloy produced by additive manufacturing; (**b**) Calculated internal friction peak area for the reverse transformation, as a function of the number of thermal cycles.

The internal friction (IF) spectra evolve on cycling as can be seen in Figure 10a. The strength of the IF peaks increases rapidly for the first few cycles, until it reaches a maximum at approximately cycle number 8. Given that the IF measures reflect the nucleation and motion of the martensite variants [97], it can be stated that the ε transformed fraction per cycle is proportional to the area of the IF peaks [65]. Then, the transformed fraction per cycle is evaluated through the integrated area of the IF peaks measured during the reverse transformation, Figure 10b, reaching a maximum of 14%.

**Figure 11:**
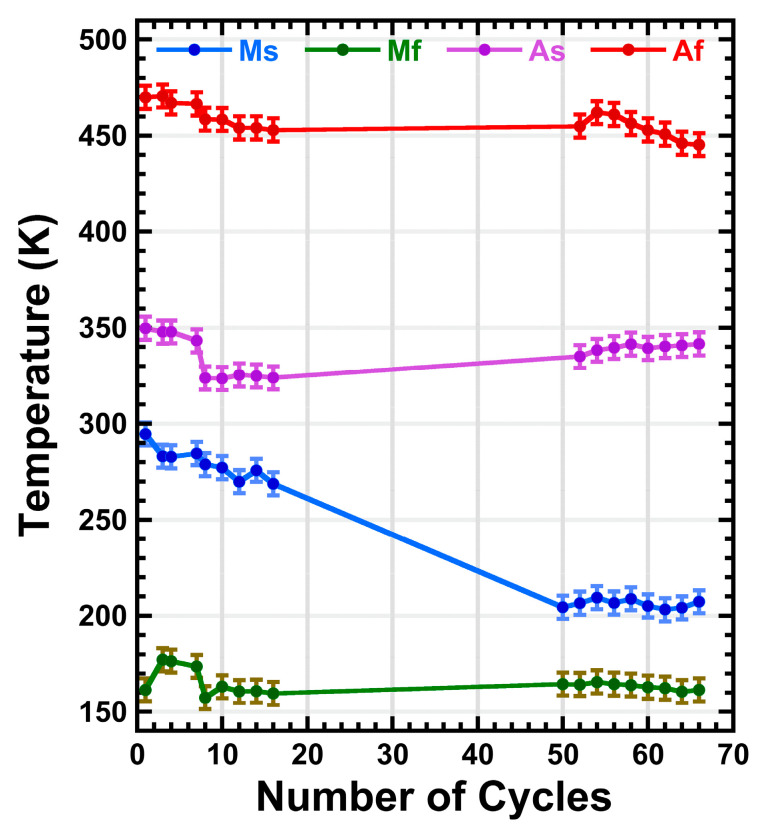
Evolution of the martensitic transformation temperatures (Ms, Mf, As, Af) for the LPBF sample presented in Figure 10.

On further cycling, the peaks smoothly decrease, just as the transformed fraction, and shift towards lower temperatures, meaning that the forward and the reverse transformations become delayed, but present an almost constant hysteresis. Finally, the ε volume fraction stabilizes around thermal cycle 60. The observed behavior in Figure 10a,b can be understood as follows. During the first cycles, the nucleation of martensite variants produces local stresses that are accommodated plastically in the matrix. This generates new dislocations in the γ structure [115], specifically in the same (111) plane as the variants that produced them, that remain through the reverse transformation [116]. As the thermal cycles add up, more nucleation points will occur and consequently, the transformed fraction rapidly grows, which explains the quick height growth of the IF peaks; a greater amount of martensite is transformed in the same temperature interval. As the density of dislocations rises, the stress field makes the nucleation of the martensite difficult and, being a non-thermoelastic alloy, exacerbates the apparition of local plastic deformation in the matrix [31] and in the γ/ε interfaces [116]. Hence, the growth of the martensite variants is hindered, resulting in a stasis mechanism in which the transformed volume fraction does not reach the 100% [117,118]. This phenomenon also leads to a delay in the MT, which agrees with the shift of the IF peaks because the transformation requires a longer overcooling to get started. Indeed, in Figure 11, the transformation temperatures, plotted as a function of the number of cycles, show a shift towards lower temperatures. Particularly, Ms undergoes a shift of almost 100 K in 50 cycles before stabilizing; the variation being slight for the Mf, Af and As temperatures. This shift of Ms can be interpreted as follows. Since the density of nucleation points grows on cycling, creating an stress field that hinders the nucleation and movement of the variants, a higher driving force is needed for the forward transformation to begin, and therefore, Ms decreases [89]. Despite the controversy on the Mf, Af and As temperature shift [118], this result has been widely observed for ternary Fe-Mn-Si [89] and quinary Fe-Mn-Si-Cr-Ni [65], including additive manufactured alloys [54].

**Figure 12:**
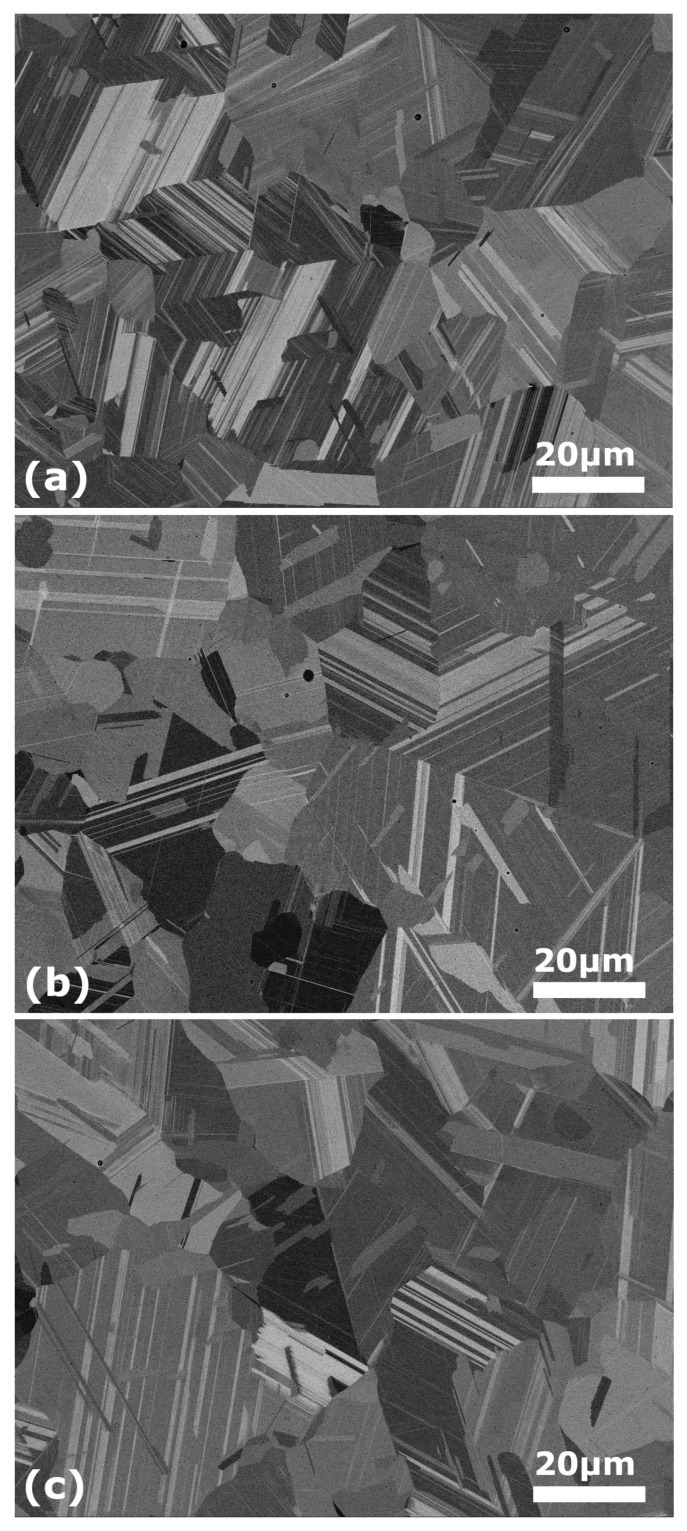
SEM (BSE) images taken for different amount of thermal cycles in order to show the martensite evolution behavior; a rapid growth during first cycles is observed, followed by a further decrease and stabilization (see Figure 10). (**a**) 3 cycles; (**b**) 11 cycles; (**c**) 51 cycles.

In parallel, to follow the evolution of the microstructure along the cycling behavior, an equivalent sample was equally cycled for its observation at SEM. Figure 12 shows the BSE images for said sample after 3 (a), 11 (b) and 51 (c) thermal cycles, respectively. The three images show the coexistence of the γ austenite matrix with the thin white plates of ε martensite. Although it is not evident at first sight, the transformed fraction is bigger for the 3 and 11 cycled samples in comparison with the 51 cycled one. This result is coherent since after a rapid growth, the highest transformed fraction was found around cycle 8. This SEM characterization confirmed the previously commented stasis phenomenon [118]; the martensite fraction never reached a fully transformed austenite. On the other hand, the observed ε martensitic transformation showed a fully reversible behavior, because no α′ martensite was created at the intersection of the ε plates on cycling. Indeed, the α′ martensite is non-reversible and has a detrimental effect on the back-movement of the ε variants during the reverse transformation [75,88]. Furthermore, from the BSE sequence it can also be appreciated that the martensite variants usually nucleate in grain boundaries and grow towards the inner side, till they reach the boundary of the opposite side. This is in agreement with previous works on similar SMA with a grain size below 30 μm [113], which is the mean grain size of our treated alloys, too.

## 5. Perspectives and Opportunities of AM in Fe-Mn-Si-Based SMA

Nowadays, the AM activity on Fe-Mn-Si-based SMA is mainly focused on the LPBF technique. In our knowledge there are no works on AM of these alloys by electron beam melting (EBM) and scarcely works using LMD [54] or other techniques like WAAM [55], for instance. Thus, in what follows, we will focus our attention in LPBF processed alloys. At present, the AM through LPBF seems to be an enough mature technology to produce compact samples (>99.9%) without pores; apparently this issue is already solved also in the case of Fe-Mn-Si-based SMA. A more relevant problem, as described in the presented results, is the compositional control and particularly the control of Mn due to the strong losses by evaporation from the melt pool. However, the progress of the evaporation control during LPBF [102] and the recent work on real time temperature monitoring during LPBF [119] allow us to be optimistic; perhaps the losses cannot be suppressed but could be well reproducible and, consequently, easy to compensate from the beginning. In what concerns to the interstitial impurities, in particular carbon and oxygen, the present work shows that in this kind of SMA the residual Al impurity becomes very useful to capture the oxygen dissolved in the austenite lattice, forming mixed (Mn,Al)_2_O_3_ oxides. The use of carbides by addition of 0.7 V and 0.2 C (wt%), has been recently employed in LPBF of Fe-Mn-Si-Cr-Ni SMA [120,121] to strengthen the austenite and increase the internal stresses. Nevertheless, no relevant improvements of the shape recovery were obtained. In our opinion, an excess of carbides fully blocks the motion of the Shockley dislocations preventing the recovery of the ε martensite. As commented above, C should not be added and the carbide formers (Nb, V, Ti) would be added only in small amounts, just to clean up the residual C in the lattice.

On the other hand, the residual internal stresses play an important role on the driving force for the recovery during the reverse transformation, as was demonstrated by adiabatic calorimetry [122] and neutron diffraction [123]. In Fe-Mn-Si-based SMA processed by AM, the internal stresses may be used to create the dislocation network required for the nucleation of the ε martensite plates, which in conventional processed samples is created by rolling. The accurate control of the melt pool temperature [119] and the cooling rates [124], should allow mastering the internal stresses and the cell size of the sub-grains during solidification [124]. Indeed, a recent work on stainless steel SS316L [125] heads for this direction. The geometrically necessary dislocations, appearing at the boundaries of the solidification cells, are arranged in a high-density dislocation network above the melt pool boundary. Another recent work [126], in the same material, shows that the change in the solidification mode may allow controlling the micro-texture of the cells. The appropriate implementation of these advanced concepts should provide a noticeable increase in the SME in Fe-Mn-Si-based SMA.

Further research and improvements are required to optimize the production of Fe-Mn-Si-based SMA through LPBF, however the opportunities are certainly there. Obviously, some applications of these SMA, like the wires or strips used to reinforce concrete [16,22], are out of the AM capabilities. Nevertheless, there are many applications, like pipe and shaft couplings, fasteners, retaining rings, self-adjustable devices, seismic dampers, etc., for which the flexibility of the AM production of SMA components by LPBF, will offer a competitive advantage respect to the conventional production technologies. Moreover, the AM through LPBF of Fe-Mn-Si-based SMA opens the door to new unforeseen applications of these smart materials.

## 6. Conclusions

The additive manufacturing of Fe-20Mn-6Si-9Cr-5Ni (wt%) shape memory alloy was approached by laser powder bed fusion (LPBF). The microstructure was studied by SEM and TEM along the different steps of the processing route, allowing the selection of the optimum post-processing thermal treatment. The additive manufactured samples exhibit a reproducible and fully reversible martensitic transformation. At the light of the obtained results, the main conclusions are summarized as follows:

- The use of pre-alloyed powders produced by gas atomization with diameters between 20 and 45 μm, allows the production of Fe-20Mn-6Si-9Cr-5Ni (wt%) shape memory alloys through additive manufacturing.
- The parameters of the additive manufacturing process, laser power and scan speed, were optimized and a remarkable 99.93% relative density was achieved by LPBF.
- The need of a further post-processing thermal treatment at 1350 K (or at least above 1273 K) was demonstrated through a detailed microstructural characterization by SEM, with EDX and EBSD and TEM, with HAADF and EDX, detectors.
- The design of the alloy composition succeeds in achieving a Neel temperature, T_N_, sufficiently low to not interact with the martensitic transformation.
- The presence of nano precipitates of (MnAl)_2_O_3_ mixed oxides has been reported in these SMA and its presence could be inherent to the additive manufacturing route of Mn-rich alloys.
- The LPBF processed and thermally treated samples exhibit a reproducible and fully reversible martensitic transformation between the γ austenite and the ε martensite. This reversibility stands over cycling, and the undesirable α′ martensite was not found.
- In the LPBF processed samples an ε transformed fraction above 14% was obtained, which is an outstanding result for a thermally induced transformation in non-strained samples.
- The cycling behavior of the ε martensite transformation, in LPBF samples, is reproducible and stable after about 60 cycles, with a fully reversible thermally transformed ε fraction above 9%.

We hope that the offered review on the state of the art in this field could be inspiring for further works on AM of Fe-Mn-Si-based SMA. It can be finally concluded that the AM by LPBF technique is a feasible methodology for elaborating Fe-Mn-Si-Cr-Ni shape memory alloys. The present work paves the way for further optimization of this technology, with regard to already existing and unforeseen new applications of these functional alloys; the new advances on AM allow us to be excitedly optimistic.

## Figures and Tables

**Table 1:** Parameters used for the different scan strategies reordered by laser power energy and scanning speed. The parameters used for the further studied samples are highlighted in green.

Num. of Experiment	Laser Power (W)	Scanning Speed (mm/s)	VED (J/mm^3^)	Compactness (%)
1	170	650	73	99.91
5	170	750	63	99.91
2	170	850	56	99.89
8	185	650	79	99.93
7	185	750	69	99.92
9	185	850	60	99.91
6	200	650	85	99.93
4	200	750	74	99.91
3	200	850	65	99.92

## Data Availability

The data presented in this study are available on request from the corresponding author. The data are not publicly available because they belong to an on going project.

## Supplementary Materials

The following supporting information can be downloaded at: https://www.mdpi.com/article/10.3390/ma16247517/s1, Figure S1: Magnetic susceptibility; Figure S2: BSE image and IPF map; Figure S3: BSE image after 1350 K treatment; Figure S4: X-ray spectrum for LPBF sample.
