# Supplementary material for: Additive Manufacturing of Fe-Mn-Si-Based Shape Memory Alloys: State of the Art, Challenges and Opportunities
Source: Materials (Basel). 2023 Dec 5;16(24):7517. doi: 10.3390/ma16247517 (PMC10744529; doi:10.3390/ma16247517)
Supplement: Supplementary file 1 [file materials-16-07517-s001.zip › materials-2752118-supplementary.pdf]

materials

Supplementary Materials

Additive Manufacturing of Fe-Mn-Si-Based Shape Memory Alloys: State of the Art, Challenges and Opportunities

Lucia Del-Río [1], Maria L. Nó [1], Raul Gómez [2], Leire García-Sesma [2], Ernesto Urionabarrenetxea [3,4], Pablo Ortega [3,4], Ane M. Mancisidor [2], Maria San Sebastian [2], Nerea Burgos [3,4] and Jose M. San Juan [1,*]

1 Department of Physics, Faculty of Science and Technology, University of the Basque Country, UPV/EHU, P.O. Box 644, 48080 Bilbao, Spain
2 LORTEK-Basque Research Technology Alliance, BRTA, Arranomendia Kalea 4A, 20240 Ordizia, Spain
3 CEIT-Basque Research Technology Alliance, BRTA, Manuel de Lardizabal 15, 20018 Donostia-San Sebastian, Spain
4 Universidad de Navarra, Tecnun, Manuel de Lardizabal 13, 20018 Donostia-San Sebastian, Spain
* Correspondence: jose.sanjuan@ehu.es; Tel.: +34-946012478

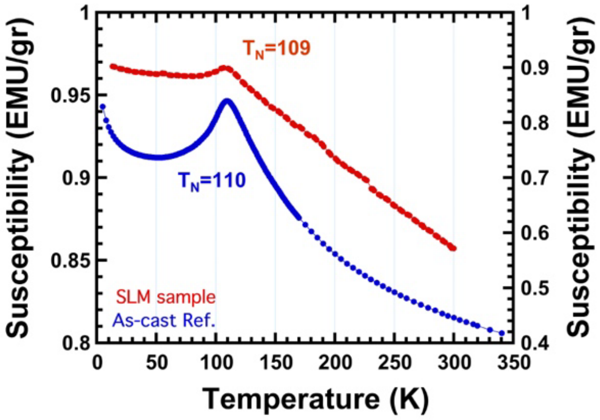

Figure S1. Magnetic susceptibility measurements to determine the Neel temperature T_N of a Fe-20Mn-6Si-.9Cr-5Ni (wt%) alloy. This feature is compared with an as-cast sample with a similar composition; Fe-16Mn-6.1Si-9.2Cr-5Ni (wt%), in order to verify whether or not the magnetic transition temperature depends on the elaboration process. The left axis corresponds to the as-cast sample (in blue), and the right axis to the SLM sample (in red). This comparison is reliable due the evaporation of the Mn during the LPBF process, losing around 2.5% of manganese content, for the selected laser power, and due to the slightly higher amount of Si and Cr of the as-cast sample, taking into account that both elements decrease the Neel temperature.

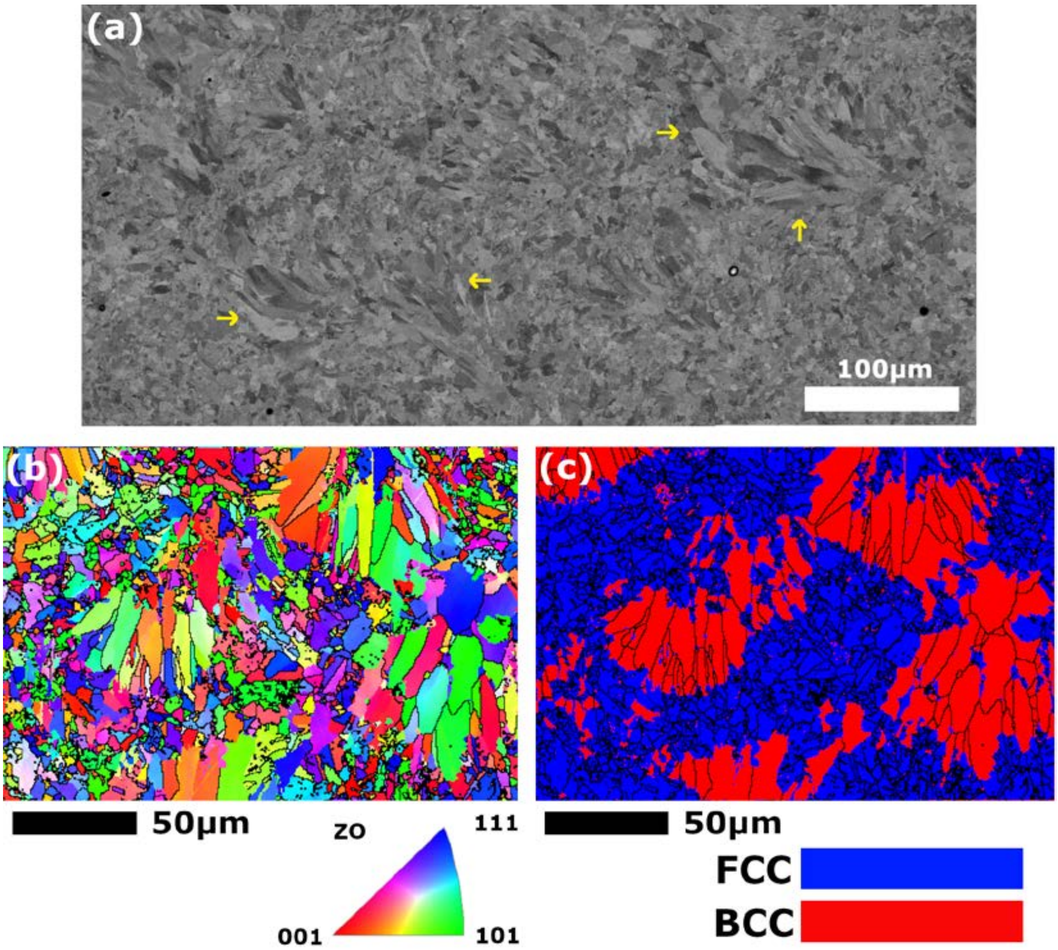

Figure S2. (a) Scanning electron microscopy BSE image in the growth-direction (YZ plane) for the unspoiled LPBF-sample with no post-process treatment; (b) Inverse pole figure (IPF) map where the orientation of the FCC and BCC grains are presented, marked by yellow arrows in (a); (c) Corresponding phase map where the FCC (blue) and BCC (red) phases are distinguished.

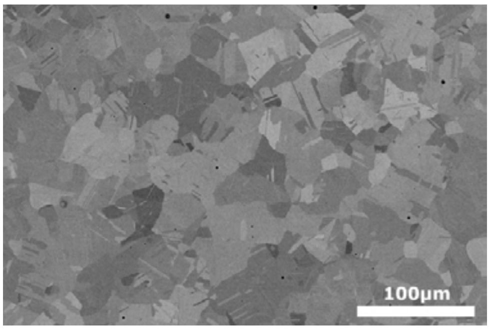

Figure S3. Scanning electron microscopy BSE image for the sample after the heat treatment at 1350 K, without any thermal cycle.

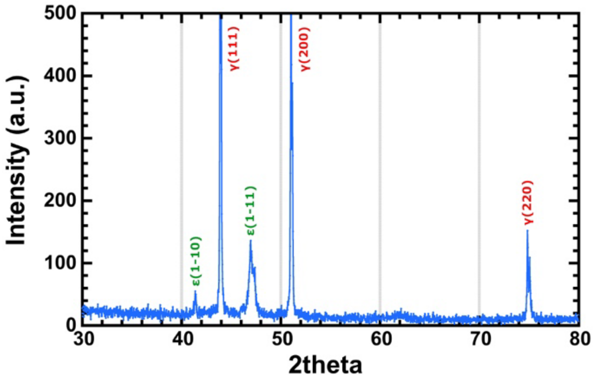

Figure S4. X-ray diffraction spectrum for the LPBF sample heat-treated at 1350K after one single thermal cycle. It is verified the coexistence of the FCC γ austenitic (red) and the HCP ε martensitic (green) phases. It also confirms that, after the proposed heat-treatment, there is no presence of BCC δ ferrite ((110) at 45º, (200) at 65º) neither BCC α' martensite ((110) at 46º). The X-ray diffraction patterns were collected by a *Philips X'Pert Pro* automatic diffractometer operating in theta-theta configuration with a secondary monochromator of Cu-K〈 radiation (λ = 1.5418 A) and a PIXcel solid state detector which has an active length in 2 θ of 3.347o. Data were collected with a step size of 0.026 and time per step of 300s; scan speed of 0.022º/s. The measurements were done at 40 kV, 40 mA and room temperature.
